# Consumo de Álcool e sua Associação com o Estilo de Vida em Indivíduos com e sem Doença Cardiovascular

**DOI:** 10.36660/abc.20250542

**Published:** 2025-11-27

**Authors:** Paulo Henrique Godoy

**Affiliations:** 1 Universidade Federal do Estado do Rio de Janeiro Rio de Janeiro RJ Brasil Universidade Federal do Estado do Rio de Janeiro, Rio de Janeiro, RJ – Brasil

**Keywords:** Consumo de Bebidas Alcoólicas, Estilo de Vida, Doenças Cardiovasculares

A associação entre o consumo de álcool e o estilo de vida é multifacetada. A quantidade e o padrão de consumo de álcool influenciam e são influenciados por outros fatores do estilo de vida, como hábitos alimentares, atividade física, tabagismo, sono, composição corporal e status socioeconômico, entre outros.^1-3^

Níveis mais elevados de consumo de álcool estão geralmente associados a comportamentos de vida menos saudáveis. O consumo excessivo de álcool é frequentemente acompanhado por aumento do consumo calórico, pior qualidade da dieta e maior prevalência de tabagismo e inatividade física.^1,2^

Por outro lado, o consumo leve a moderado de álcool é mais frequentemente observado entre indivíduos com estilos de vida mais saudáveis, incluindo melhores padrões alimentares, menor índice de massa corporal, maior adesão à atividade física e menores taxas de tabagismo em comparação com aqueles com alto consumo de álcool.^3,4^

Esses comportamentos mais saudáveis entre bebedores moderados podem confundir as associações observadas entre o consumo moderado de álcool e a redução do risco de certas doenças crônicas, como diabetes tipo 2 e doenças cardiovasculares (DCV). Quando as análises são ajustadas para esses fatores de estilo de vida, as aparentes associações protetoras do consumo moderado de álcool podem ser atenuadas ou consideradas não significativas. Isso sugere que os benefícios observados podem ser atribuídos a comportamentos de estilo de vida (CEV) mais saudáveis, mais comuns entre bebedores moderados, e não ao álcool em si.^3,4^

Portanto, é importante reconhecer que a interação entre o consumo de álcool e os CEV é complexa, e possíveis fatores de confusão relacionados ao estilo de vida devem ser cuidadosamente considerados ao interpretar associações entre o consumo de álcool e os resultados de saúde.

A relação entre o consumo de álcool e as DCV também é complexa e permanece controversa. O uso nocivo de álcool por indivíduos com doença cardíaca (DC) está associado a um aumento do risco cardiovascular, morbidade e mortalidade.^5,6^ Por outro lado, se tem levantado a hipótese de que o consumo leve a moderado de álcool pode conferir efeitos cardioprotetores, reduzindo o risco de várias formas de DCV.^6^ Embora tal consumo possa parecer cardioprotetor em comparação com a abstinência, esse benefício provavelmente é confundido por fatores de estilo de vida e pode não ser suficiente para recomendar o consumo de álcool para proteção cardiovascular – especialmente entre pacientes com DCV estabelecida.^7-9^

A interação entre o consumo de álcool e os CEV influencia a progressão das DCV, particularmente em populações vulneráveis.^10^ A avaliação de risco do consumo de álcool entre indivíduos com DCV pode variar significativamente com base em seus perfis de estilo de vida e fatores genéticos, incluindo padrões de consumo e estado geral de saúde.^10,11^Conforme observado anteriormente, mesmo entre bebedores leves a moderados, o benefício potencial pode variar e ser influenciado por fatores individuais de estilo de vida.^8,9^

Nesse contexto, o estudo de Martinelli-Filho et al., intitulado " Consumo Abusivo de Álcool em Cardiopatas: Análise de Risco pelo Comportamento de Estilo de Vida – Projeto PROSA (Projeto Saúde e Álcool)",^12^ publicado nesta edição, analisou a associação entre CEV e consumo nocivo de álcool (CNA) em indivíduos com e sem DC. Foi realizado um estudo de coorte prospectivo ao longo de três anos, incluindo duas coortes distintas: uma composta por pacientes ambulatoriais clinicamente estáveis com DC e a outra por voluntários recrutados entre familiares ou cuidadores de pacientes da coorte de DC, bem como funcionários do hospital.

O consumo de álcool foi avaliado usando o Teste de Identificação de Distúrbios por Uso de Álcool (AUDIT), e as variáveis relacionadas ao estilo de vida incluíram tabagismo, sobrepeso/obesidade, ansiedade, depressão, inatividade física, nível educacional e renda. Além disso, um modelo preditivo para CNA foi desenvolvido com base nas variáveis CEV. Entre uma amostra total de 4.080 indivíduos, 1.999 foram incluídos na coorte de DC e 2.081 na coorte de voluntários. Os autores relataram a identificação dos mesmos preditores independentes de CNA em ambas as coortes - sexo masculino, idade, comportamento de estilo de vida regular e comportamento de estilo de vida não saudável. O modelo mostrou que a melhora no CEV em direção a um estado mais saudável foi associada à redução do CNA após três anos de acompanhamento. Uma tendência semelhante foi observada quando o CEV piorou, em relação ao CNA.

Apesar de suas limitações, o presente estudo fornece insights valiosos sobre a associação entre o CNA e fatores de estilo de vida em indivíduos com e sem DC. O uso nocivo de álcool, quando associado a fatores adversos de estilo de vida, pode influenciar o risco cardiovascular e outras doenças em ambos os grupos. Indivíduos com transtorno por uso de álcool apresentam maior incidência de DCV em comparação com aqueles sem o transtorno.^13^ A compreensão desses fatores é crucial para o desenvolvimento de estratégias eficazes de prevenção e tratamento.

Dado o nível atual de evidências, ainda há conhecimento insuficiente para sustentar o consumo de álcool como parte de um estilo de vida saudável. Em particular, para indivíduos com doenças cardíacas, as diretrizes clínicas atuais não recomendam o incentivo ao consumo de álcool para proteção cardiovascular e reconhecem o potencial de interações entre álcool e medicamentos. Em vez disso, se devem enfatizar CEV saudável bem estabelecidos, como atividade física regular, adesão a uma dieta nutritiva, manutenção de um peso corporal saudável e cessação do tabagismo.^5,8^
