# Supplementary material for: Consumo de Álcool e sua Associação com o Estilo de Vida em Indivíduos com e sem Doença Cardiovascular
Source: Arq Bras Cardiol. 2025 Nov 27;122(10):e20250542. [Article in Portuguese] doi: 10.36660/abc.20250542 (PMC12671687; doi:10.36660/abc.20250542)
Supplement: Translation

# Translation: Alcohol Consumption and Its Association with Lifestyle in Individuals with and without Cardiovascular Disease

**DOI:** 10.36660/abc.20250542i

**Authors:** Paulo Henrique Godoy

**Affiliations:** 1 Rio de Janeiro RJ Brazil Universidade Federal do Estado do Rio de Janeiro, Rio de Janeiro, RJ – Brazil

**Keywords:** Alcohol drinking, Life Style, Cardiovascular Diseases

The association between alcohol consumption and lifestyle is multifaceted. The amount and pattern of alcohol intake both influence and are influenced by other lifestyle factors, such as dietary habits, physical activity, smoking, sleep, body composition, and socioeconomic status, among others.^1-3^

Higher levels of alcohol consumption are generally associated with less healthy lifestyle behaviors (LSB). Excessive alcohol intake is frequently accompanied by increased caloric consumption, poorer dietary quality, and a higher prevalence of smoking and physical inactivity.^1,2^

Conversely, light to moderate alcohol consumption is more often observed among individuals with healthier lifestyles, including better dietary patterns, lower body mass index, greater adherence to physical activity, and lower smoking rates compared to those with high alcohol intake.^3,4^

These healthier behaviors among moderate drinkers may confound the observed associations between moderate alcohol intake and reduced risk of certain chronic diseases, such as type 2 diabetes and cardiovascular disease (CVD). When analyses are adjusted for these lifestyle factors, the apparent protective associations of moderate alcohol consumption may be attenuated or rendered non-significant. This suggests that the observed benefits may be attributable to healthier LSB more common among moderate drinkers rather than to the alcohol itself.^3,4^

Therefore, it is important to recognize that the interaction between alcohol intake and LSB is complex, and potential confounding by lifestyle-related factors must be carefully considered when interpreting associations between alcohol consumption and health outcomes.

The relationship between alcohol consumption and CVD is also complex and remains controversial. Harmful alcohol use in individuals with heart disease is associated with increased cardiovascular risk, morbidity, and mortality.^5,6^ Otherwise, it has been hypothesized that light to moderate alcohol intake may confer cardioprotective effects, reducing the risk of various forms of CVD.^6^ Although such consumption might appear cardioprotective compared to abstinence, this benefit is likely confounded by lifestyle factors and may not be sufficient to recommend alcohol consumption for cardiovascular protection - especially among patients with established CVD.^7-9^

The interaction between alcohol consumption and LSB influences the progression of CVD, particularly in vulnerable populations.¹¹ The risk assessment of alcohol intake among individuals with CVD may vary significantly based on their lifestyle profiles and genetic factors, including patterns of consumption and overall health status.^10,11^ As noted earlier, even among light to moderate drinkers, the potential benefit may vary and be influenced by individual lifestyle factors.^8,9^ In this context, the study by Martinelli-Filho et al., titled "Harmful Alcohol Consumption in Patients with Heart Disease: A Risk Analysis by Lifestyle Behavior – The Brazilian PROSA Project (Projeto Saúde e Álcool),"^12^ published in this issue, analyzed the association between LSB and harmful alcohol consumption (HAC) in individuals with and without heart disease. A prospective cohort study was conducted over three years, including two distinct cohorts: one comprising clinically stable outpatients with heart diseases (HD), and the other consisting of volunteers recruited among family members or caregivers of patients in the HD cohort, as well as hospital staff.

Alcohol consumption was assessed using the Alcohol Use Disorders Identification Test (AUDIT), and lifestyle-related variables included smoking, overweight/obesity, anxiety, depression, physical inactivity, educational level, and income. Additionally, a predictive model for HAC was developed based on LSB variables. Among a total sample of 4,080 individuals, 1,999 were included in the heart disease cohort and 2,081 in the volunteer cohort. The authors reported identifying the same independent predictors of HAC across both cohorts - male sex, age, regular lifestyle behavior, and unhealthy lifestyle behavior. The model showed that improvement in LSB toward a healthier status was associated with reduced HAC after three years of follow-up. A similar trend was observed when LSB worsened, in relation to HAC.

Despite its limitations, the present study provides valuable insights into the association between HAC and lifestyle factors in individuals with and without heart disease. Harmful alcohol use, when associated with adverse lifestyle factors, may influence cardiovascular and other disease risk in both groups. Individuals with alcohol use disorder show a higher incidence of CVD compared to those without the disorder.^13^ Understanding these factors is crucial for developing effective prevention and management strategies.

Given the current level of evidence, there is still insufficient knowledge to support alcohol consumption as part of a healthy lifestyle. In particular, for individuals with heart disease, current clinical guidelines do not recommend encouraging alcohol intake for cardiovascular protection and acknowledge the potential for alcohol-medication interactions. Instead, emphasis should be placed on well-established healthy LSB, such as regular physical activity, adhering to a nutritious diet, maintaining a healthy body weight, and smoking cessation.^5,8^
